# The interaction between metabolic rate, habitat choice, and resource use in a polymorphic freshwater species

**DOI:** 10.1002/ece3.9129

**Published:** 2022-07-31

**Authors:** Matilda L. Andersson, Kristin Scharnweber, Peter Eklöv

**Affiliations:** ^1^ Department of Ecology and Genetics Uppsala University Uppsala Sweden; ^2^ Department of Aquatic Sciences and Assessment Swedish University of Agricultural Sciences Uppsala Sweden; ^3^ Department of Plant Ecology and Nature Conservation University of Potsdam Potsdam Germany

**Keywords:** intraspecific variation, metabolic rate, morphometrics, *Perca fluviatilis*, plasticity, resource use, respirometry, stable isotopes

## Abstract

1. Resource polymorphism is common across taxa and can result in alternate ecotypes with specific morphologies, feeding modes, and behaviors that increase performance in a specific habitat. This can result in high intraspecific variation in the expression of specific traits and the extent to which these traits are correlated within a single population. Although metabolic rate influences resource acquisition and the overall pace of life of individuals it is not clear how metabolic rate interacts with the larger suite of traits to ultimately determine individual fitness.
2. We examined the relationship between metabolic rates and the major differences (habitat use, morphology, and resource use) between littoral and pelagic ecotypes of European perch (*Perca fluviatilis)* from a single lake in Central Sweden.
3. Standard metabolic rate (SMR) was significantly higher in pelagic perch but did not correlate with resource use or morphology. Maximum metabolic rate (MMR) was not correlated with any of our explanatory variables or with SMR. Aerobic scope (AS) showed the same pattern as SMR, differing across habitats, but contrary to expectations, was lower in pelagic perch.
4. This study helps to establish a framework for future experiments further exploring the drivers of intraspecific differences in metabolism. In addition, since metabolic rates scale with temperature and determine predator energy requirements, our observed differences in SMR across habitats will help determine ecotype‐specific vulnerabilities to climate change and differences in top‐down predation pressure across habitats.

## Graphical abstract

This manuscript presents new insights into the patterns of intraspecific variation in metabolic rates and how these are correlated with other adaptive traits in a polymorphic species. We show that two ecotypes of European perch living in adjacent habitats with no observed barriers to movement or reproduction have significantly different standard metabolic rates (SMR) and aerobic scopes (AS). We suggest that metabolic rates should be included in the larger suite of traits examined in studies of polymorphic species and that the observed differences can help determine under which conditions each ecotype can persist.

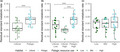

## 1 INTRODUCTION

Metabolic rates determine an individual's cost of living, and as such, differences between individuals' or populations' metabolic rates can determine their fitness and ultimately success in a given environment (Hulbert & Else, 2000). Studies on polymorphic species often focus on intraspecific variation in diet, morphology, and behavior and how these traits can provide the competitive advantage that allows an individual or a group of individuals to exploit a specific niche or habitat (Hayden et al., 2014; Skúlason et al., 2019; Skúlason & Smith, 1995). Including metabolic rates as part of this larger suite of traits is comparatively rare, even though differences in metabolic rates could impact individual fitness as much as other aspects of the phenotype (but see Rouleau et al. (2010) and Bergstrom et al. (2019)). Like morphological traits, specific metabolic phenotypes can be related to genetic differences, but also display a degree of plasticity that helps individuals persist under variable conditions (Norin & Metcalfe, 2019; Svanbäck & Eklöv, 2006). In intraspecific comparisons, high metabolic rates have proved beneficial for active animals living in structurally simple habitats (Reid et al., 2012; Seibel & Drazen, 2007) and high predation environments (Auer et al., 2018). These environments also elicit changes in morphology and activity and correspond with differences in diet, suggesting that some of the same drivers may cause the differences in metabolic rates (Olsson & Eklöv, 2005). Metabolic rates can also differ between sexes (Ducret et al., 2020; Ladds et al., 2017; Madenjian, 2011). For example, males with costly secondary sexual characteristics or high activity rates, which are both subject to strong selection pressure, often have higher metabolic rates than females (Henderson et al., 2003; Makiguchi et al., 2017; Somjee et al., 2018).

Standard metabolic rate (SMR) is the minimum oxygen consumption rate (ṀO_2_) needed to sustain an animal, while maximum metabolic rate (MMR) is the maximum ṀO_2_ an individual can achieve. Aerobic scope (AS) is calculated as the difference between SMR and MMR and is a measure of an individual's capacity to perform oxygen‐consuming functions such as movement, digestion, growth, and reproduction (Auer et al., 2015b; Clark et al., 2013; Eliason et al., 2011). These rates scale with body mass, but even controlling for this and other confounding factors, metabolic rates can still vary up to three‐fold between individuals (Burton et al., 2011). Some of this variation may be explained by morphological differences such as the cost of transport associated with increased hydrodynamic drag (Boily & Magnan, 2002; Pettersson & Brönmark, 1999), while some can be explained by environmental differences during development or differences in personality (Burton et al., 2011). Metabolic rates correspond with different aspects of an individual's life history, and by examining these traits within a single population of a well‐studied polymorphic species, we can begin to understand how differences in metabolic rates may correspond with other previously observed differences between ecotypes.

In order to examine whether metabolism is part of a larger suite of correlated traits that diverge across habitats, we used European perch (*Perca fluviatilis*), a widespread polymorphic freshwater fish. Similar to other polymorphic fish that differentiate along the littoral‐pelagic axis, littoral perch inhabit shallow, vegetated nearshore areas and typically have deeper bodies and downturned mouths, which allows them to navigate complex habitats, while pelagic perch are found in open water and are more streamlined to facilitate their active foraging style optimized for pelagic prey (Bourke et al., 1999; Robinson & Wilson, 1994; Svanbäck & Eklöv, 2003, 2004). Using a combination of stomach content and stable isotope analyses, past studies on perch have also found differences in each ecotype's niche width and individual specialization related to their habitat use (Chaguaceda et al., 2020; Marklund et al., 2019). Pelagic perch have high pelagic resource reliance and a narrow niche, while littoral perch have lower pelagic resource reliance on average but a wide niche resulting from a subset of littoral individuals that live in the littoral but forage in the pelagic (Bartels et al., 2016; Marklund et al., 2019). Based on this pattern, we can use carbon stable isotope values to group fish by their pelagic resource use (high, mid, and low) or resource use in combination with habitat (littoral‐low, littoral‐high, and pelagic‐high) to examine whether there is a relationship between resource use and metabolic rates in our system. Using these groupings, combined with geometric morphometrics and respirometry, allowed us to explore the connection between metabolism and morphology, habitat use, and resource use between ecotypes. We predict that pelagic perch will have higher metabolic rates on average because of their active foraging style, but that when controlling for habitat, deeper‐bodied individuals will have a lower SMR to compensate for the higher swimming costs associated with their morphology. We also expect that fish living in the littoral but feeding in the pelagic will have intermediate SMRs reflecting their littoral habitat use but pelagic resource use. Since MMR tends to show less plasticity (Auer et al., 2016; Sandblom et al., 2016), we do not expect that MMR will covary with resource use or morphology and that AS will therefore respond similarly to SMR.

## 2 MATERIALS AND METHODS

All fish collection and experiments were performed under evaluation and permission from the Uppsala authority for ethics of animal experimentation (ethics license #C59/15).

### 2.1 Fish collection and husbandry

Perch were collected between August 21 and 28, 2018, from lake Erken (59°50′09.6”N, 18°37′52.3″E), a mid‐sized (23.7 km^2^) mesotrophic lake in Central Sweden. Littoral perch were caught in ~2 m deep vegetated areas along the shoreline, and pelagic perch were caught in the top 5 m of the pelagic zone, 200–500 m from shore, in an area ~9 m deep. All fish were caught via angling with barbless hooks during daylight hours. The fish were transported to the Uppsala University aquarium facility in cooled and aerated boxes. Following transport, individuals were anesthetized using 60 mg L^−1^ benzocaine, weighed (g), tagged with colored elastomer (Northwest Marine Technology Inc.) at the base of their caudal fin, and photographed on their left side. 103 perch (53 littoral and 50 pelagic) were tagged and photographed for morphometrics since this technique is known to be sensitive to small sample size. ṀO_2_ and isotope composition was measured in a subset of these fish (littoral: *n* = 22, 140 ± 17 mm (mean ± standard deviations [SD]), 27.7 ± 9.7 g, pelagic: *n* = 29, 141 ± 17 mm, 26.9 ± 9.3 g). Individuals were housed with similar‐sized conspecifics in 105 L (75 × 40 × 35 cm), flow‐through aquaria with the bottom covered by a 3‐cm thick layer of sand, and fed to satiation daily with frozen chironomids (Ruto Frozen Fishfood, Montfort, the Netherlands) until respirometry trials began. The lab had a 16‐h light (L): 8‐h dark (D) cycle and the water temperature was maintained at 18 ± 0.5°C (mean ± SD). Aside from the stress experienced during the chase designed to elicit MMR, stress was minimized throughout the experiment. Fish were sacrificed following respirometry trials using an overdose of benzocaine. We weighed and measured sacrificed fish to the nearest 0.01 g and 0.01 mm, respectively, and dissected a sample of dorsal muscle tissue for stable isotope analyses.

### 2.2 Geometric Morphometrics

Perch were photographed on the left side, and 16 landmarks on each fish were digitized using TPS‐dig2 (see Bartels et al., 2012 for placement of landmarks). Digitized landmark data were imported into MorphoJ (Klingenberg, 2011) for all analyses. The data were checked for outliers and corrected for body size by regressing the Procrustes coordinates on centroid size, and the residuals from this regression were used for both the discriminant function analysis (DFA) and principal component analyses (PCA) (Klingenberg, 2016). We used the DFA to show general morphological differences between our littoral and pelagic perch. However, because DFAs are designed to describe morphological differences between predetermined groups, we used PC scores as the measures of morphology within our models since it describes differences between individuals (Zelditch et al., 2004). As is often observed, PC1 primarily explained bending, an unwanted artifact from the photographing process (Siwertsson et al., 2013; Valentin et al., 2008). We thus used PC2 and PC3 instead.

### 2.3 Respirometry setup

ṀO_2_ was measured using an intermittent flow respirometry system (Loligo Systems, Viborg, Denmark). A detailed description of the respirometry setup is described in Andersson et al. (2020). Briefly, our system comprised of four acrylic respirometry chambers (size‐matched to each fish), which were submerged in two aquaria (two chambers per tank) containing air‐stones to maintain oxygen at air‐saturation levels and attached to an external heating bath which maintained the water temperature at 18.1 ± 0.10°C (mean ± SD). Water was recirculated between the two aquaria through a UV filter to reduce background respiration in the system caused by bacterial growth. Oxygen concentration was measured using a Wiltrox4 oxygen meter (LoligoSystems), and water temperature and phase length were controlled using AutoResp software meter (LoligoSystems). Measurement loops consisted of a 180 s flush phase, a 30 s wait phase, and a 210 s measurement phase. Following each trial, the system was cleaned with ~15 ml bleach and flushed with fresh water to prevent the build‐up of microbes. Following cleaning, the aquaria were refilled with tap water that had been aerated and maintained at 18 ± 0.5°C for a minimum of 24 h.

### 2.4 Measurements of metabolic rates

Fish were fasted for approximately 24 h prior to the start of each trial. To elicit maximum metabolic rate, fish were manually chased with a hand net for 3 minutes in a circular arena (diameter = 50 cm, water depth = 12 cm) filled with aerated tap water at 18 ± 0.1°C (Brijs et al., 2015; Sandblom et al., 2016; Svendsen et al., 2016). Although a subsequent study found that a chase protocol results in a lower MMR than allowing the fish to reach MMR through spontaneous activity (Andersson et al., 2020), our focus here is on a comparison of MMR and not the magnitude itself, so we expect that the chase protocol did not impact our results. Following the chase, individuals were removed from the arena, transported in a bucket filled with aerated water (~5 s), and placed in the respirometer chamber during the “wait” phase. The wait phase is necessary to account for a lag in the system, which can result in a non‐linear oxygen curve (Loligo‐Systems, 2020), and fish were placed in the chamber during this phase so that the first measurement phase would be linear and thereby included in the analysis. Fish remained in the respirometry chambers in a dark room overnight for a minimum of 14 h (121 measurement loops).

Chamber‐specific background respiration was calculated by fitting linear regressions between measurements of background respiration with *R* ^2^ > 0.1, taken immediately before and after each trial. Each trial was adjusted for background respiration by subtracting fitted values estimating background respiration from measures of non‐mass specific ṀO_2_ (mgO_2_ h^−1^) for each fish at each timepoint. Any estimates of ṀO_2_ with an *R* ^2^ < 0.90 were removed prior to calculations of SMR, MMR, and AS. SMR was calculated as the mean of the lowest 10% of ṀO_2_ measures, MMR as the global maximum ṀO_2_, and we calculated both absolute aerobic scope (AAS) (MMR ‐ SMR) and factorial aerobic scope (FAS) (MMR/SMR) (Andersson et al., 2020). We log transformed body mass and metabolic rate measures to normalize the data and correct for skew associated with metabolic data. Using the transformed values, we performed a linear regression of each metabolic measure against body mass and used the residuals from these models in our analyses in order to account for the effect of mass on metabolic rates (Auer et al., 2015a).

### 2.5 Stable isotope analysis

Zooplankton for the pelagic stable isotope baseline was collected using a Ø25 cm plankton net with 60 μm mesh size via vertical plankton tows in the pelagic zone from 1 m above the sediment to the surface and by towing the plankton net along the surface of the pelagic zone behind the boat. The adductor muscle from mussels was also used as a pelagic baseline since mussels are long lived filter‐feeders with low turnover rates, making them less sensitive to temporal variation in pelagic resources and a good baseline for pelagic fish (Post, 2002; Vuorio et al., 2007).

Benthic invertebrates for the benthic stable isotope baseline were collected from shallow littoral areas using a kick net and were sorted by species and kept in tap water overnight to allow for gut evacuation. Zooplankton and invertebrates were then stored at −20 °C until sample processing. Dissected fish muscle tissue, zooplankton, and benthic invertebrates were dried in a drying oven at 60°C for 48 h. Dried samples were ground to a fine powder using a mortar and pestle, and approximately 1 mg of powder was transferred to tin capsules for analysis. Stable isotope analysis of carbon and nitrogen on perch tissue and invertebrate samples were conducted at the Stable Isotope Facility of University of California, Davis, California, USA using a PDZ Europa ANCA‐GSL elemental analyzer interfaced to a PDZ Europa 20–20 isotope ratio mass spectrometer (Sercon). Samples were not lipid corrected as C:N ratio was low (3.22 ± 0.04, mean ± SD) (Kiljunen et al., 2006). Results are expressed using δ notation on the international standards VPBD (Pee Dee Belemnite) and AIR (Ambient Inhalable Reservoir) for δ^13^C and δ^15^N, respectively. Five percent of perch samples were processed in duplicate, and measurement error was 0.02 ‰ for δ ^13^C and 0.05 ‰ for δ ^15^N.

### 2.6 Statistical analysis

We used a k‐means cluster analysis using the cluster package (Maechler et al., 2019) to split the fish into categories based on their pelagic resource use, using nstart = 25. Nstart specifies how many times the clustering algorithm is run with random starts and Flynt and Dean (2016) recommend using at least nstart = 10. For our combined habitat+resource use condition, we used a cluster analysis based on δ^13^C values with two centers, which split fish into high and low pelagic resource use groups. We used catch location and cluster assignment to create four groups, Littoral‐low (*n* = 12), Littoral‐high (*n* = 10), Pelagic‐low (*n* = 1), and Pelagic‐high (*n* = 27) based on the method used by Chaguaceda et al. (2020). The Pelagic‐low group was excluded from analyses due to small sample size. For our resource use condition, we used a cluster analysis based on δ^13^C values with three centers, which split fish into low (*n* = 10), moderate (*n* = 16), and high (*n* = 24) pelagic resource use categories. The habitat‐only condition had two groups, littoral (*n* = 22) and pelagic (*n* = 29).

We used multiple linear regression to analyze the effect of morphology, habitat, resource use, and sex on our four focal metabolic measures (SMR, MMR, AAS, and FAS). In our models that include morphology and habitat, we used an interaction term between the PC score and habitat to check whether the effect of morphology on the metabolic rate differed between the habitats. We used an ANOVA with type 3 sums of squares for models with an interaction and type 2 sums of squares for models with no interaction. All models met the assumptions of normality and equal variance, and though there are outliers, none exceeded a cook's distance of 0.5. We did pair‐wise comparisons on the estimated marginal means with Tukey adjustment, using the emmeans package (Russell, 2020), to determine whether there were significant differences between diet groups. Respirometry data was imported using package rMR (Moulton, 2018), and plots were created using ggplot2 (Wickham, 2016). All analyses were performed in R version 4.0.2 (R Core Team, 2020).

## 3 RESULTS

**FIGURE 1:**
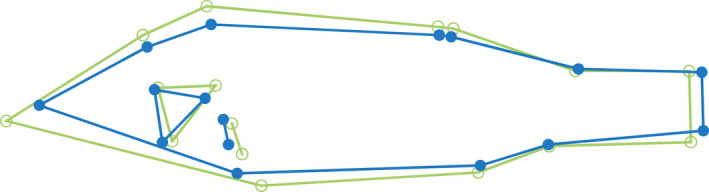
Visualization (with 4x magnification) of the morphological differences between littoral (green, open circles) and pelagic (blue, closed circles) perch from lake Erken, based on a discriminate function analysis (DFA).

**FIGURE 2:**
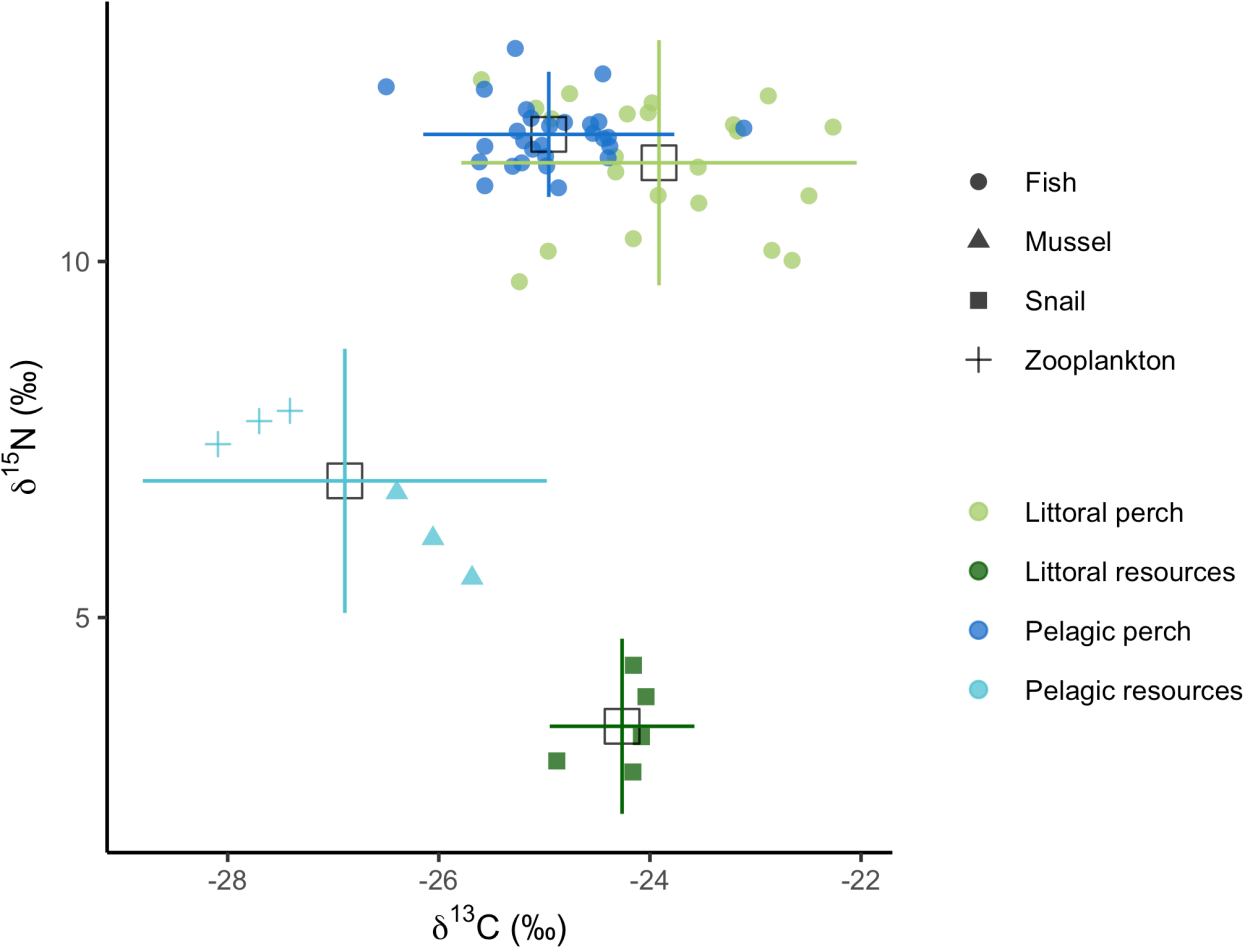
Stable isotope bi‐plot showing the mean (± standard deviation) and individual δ^13^C and δ^15^N stable isotope values of each perch in addition to littoral and pelagic primary consumers.

**FIGURE 3:**
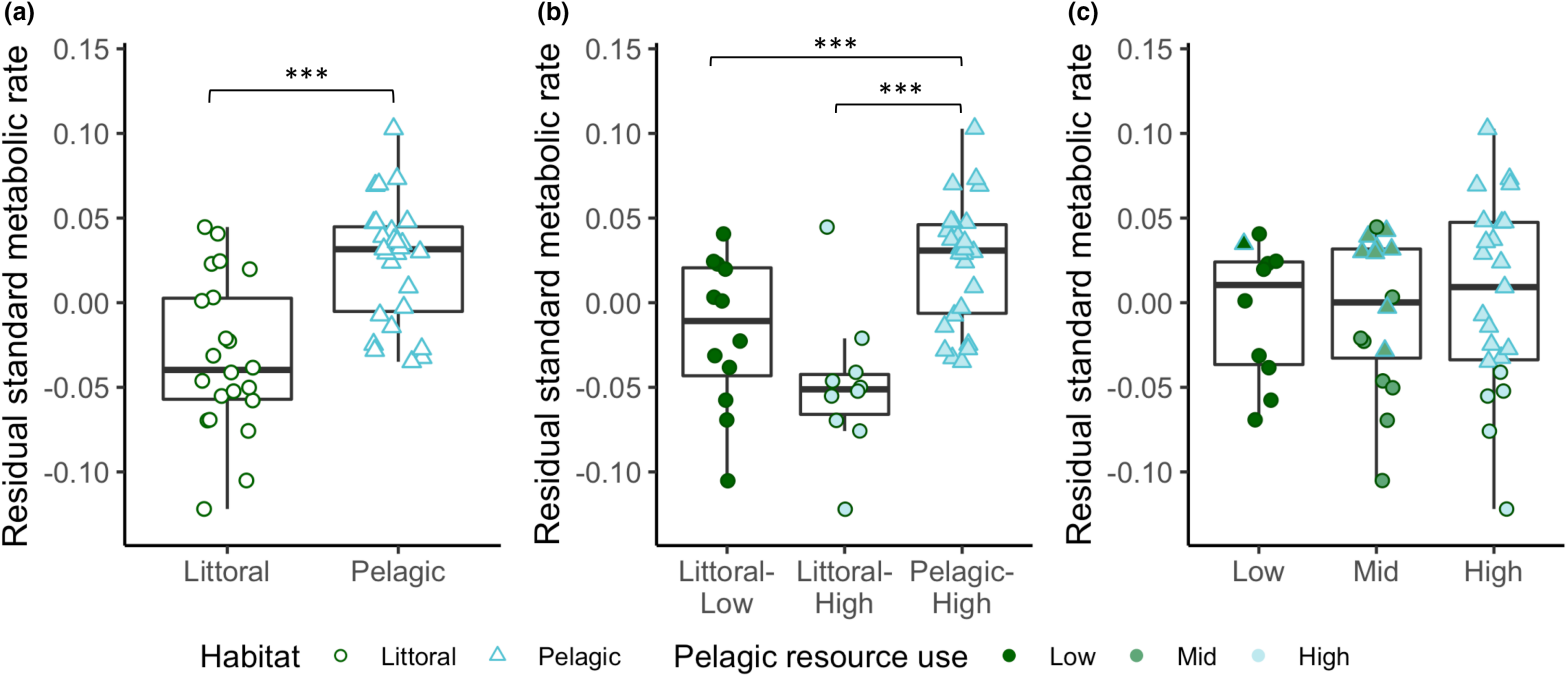
Residual standard metabolic rate (mg O_2_ h^−1^) across (a) habitat, (b) habitat + resource use, and (c) resource use groups in perch. Asterisks indicate significant differences (*p < .05, **p < .01, ***p < .001) based on pairwise comparisons controlling for sex. Boxplots depict median, 25th and 75th percentile, and whiskers extend to the maximum and minimum values with the exception of plots with outliers (>1.5 * interquartile range) which are represented by dots.

**FIGURE 4:**
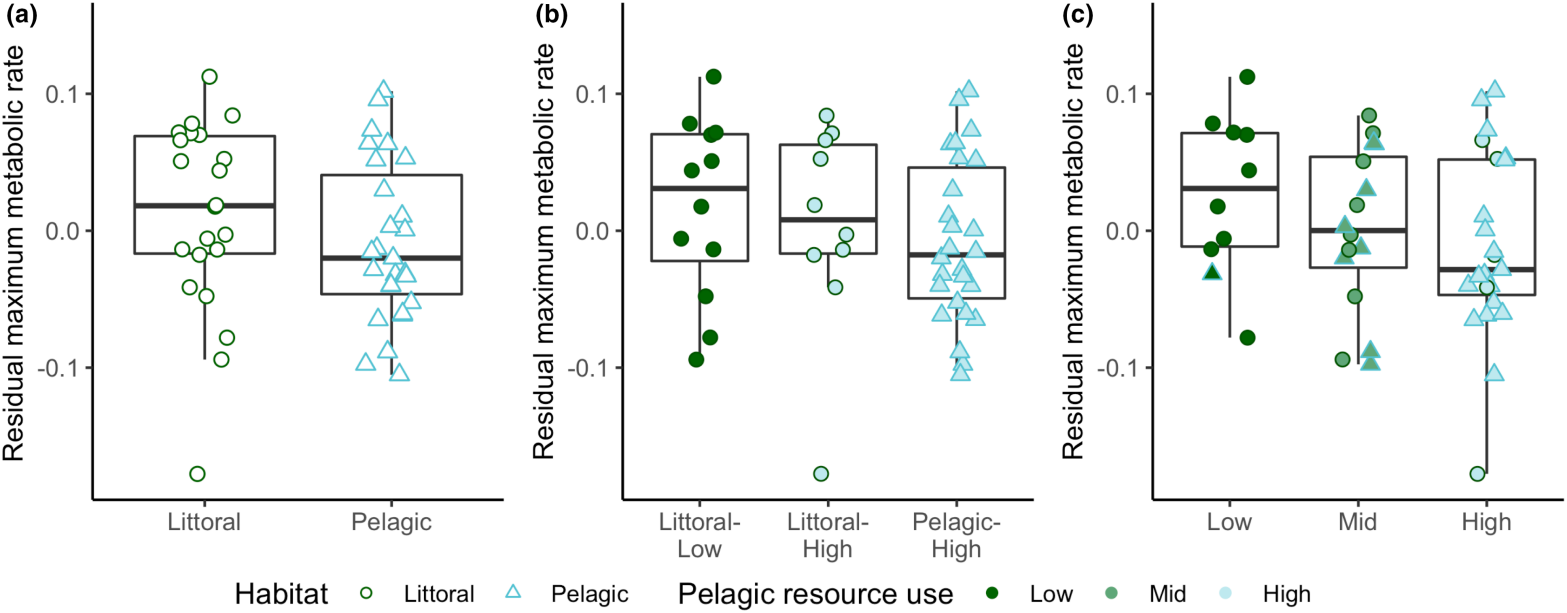
Residual maximum metabolic rate (mg O_2_ h^−1^) across (a) habitat, (b) habitat + resource use, and (c) resource use groups in perch. There were no significant differences based on pairwise comparisons controlling for sex. Boxplots depict median, 25th and 75th percentile, and whiskers extend to the maximum and minimum values with the exception of plots with outliers (>1.5 * interquartile range) which are represented by dots.

**FIGURE 5:**
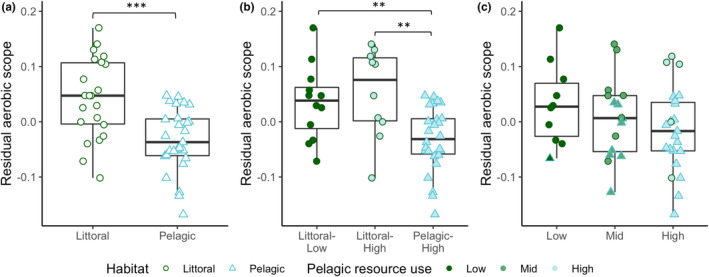
Residual factorial aerobic scope (mg O_2_ h^−1^) across (a) habitat, (b) habitat + resource use, and (c) resource use groups in perch. Asterisks indicate significant differences (*p < .05, **p < .01, ***p < .001) based on pairwise comparisons controlling for sex. Boxplots depict median, 25th and 75th percentile, and whiskers extend to the maximum and minimum values with the exception of plots with outliers (>1.5 * interquartile range) which are represented by dots.

**FIGURE 6:**
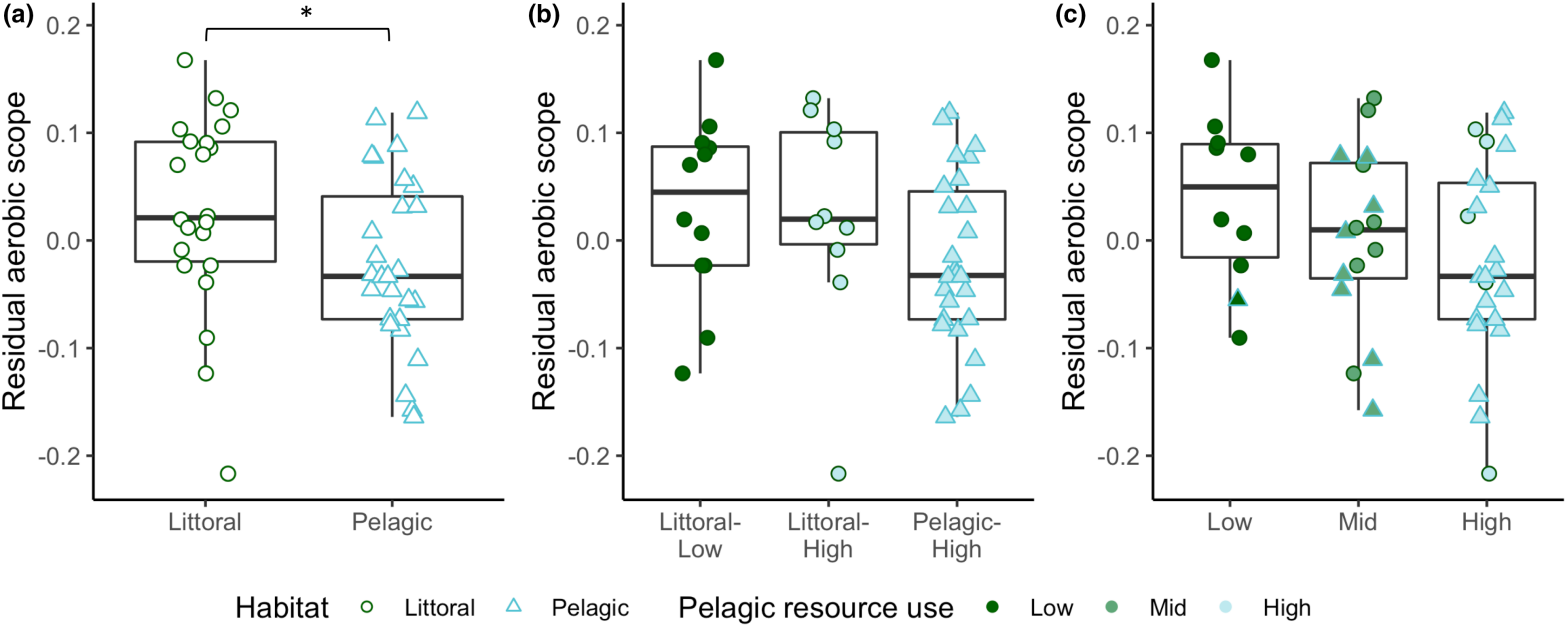
Residual absolute aerobic scope (mg O_2_ h^−1^) across of (a) habitat, (b) habitat + resource use, and (c) resource use groups in perch. Asterisks indicate significant differences (*p < .05, **p < .01, ***p < .001) based on pairwise comparisons controlling for sex. Boxplots depict the median, 25th^,^ and 75th percentile, and whiskers extend to the maximum and minimum values except for plots with outliers (>1.5 * interquartile range) which are represented by dots.

The DFA correctly assigned 82% of individuals into their respective habitats, and there were clear differences between the littoral and pelagic ecotypes (Mahalanobis distance D = 3.49, *p* < .001) (Figure S1). The two ecotypes showed the expected divergence with littoral perch exhibiting a deeper body, sub‐terminal mouth, and robust caudal peduncle, while the pelagic perch have a more terminal mouth and fusiform body (Figure 1). As shown in previous studies, pelagic perch have significantly lower δ^13^C values than littoral perch (−24.96 ± 0.61 and − 23.91 ± 0.96 (average ± SD), respectively; U = 490, *p* < .001) (Chaguaceda et al., 2020; Quevedo et al., 2009; Scharnweber, Strandberg, Marklund, & Eklöv, 2016). The δ^13^C–δ^15^N biplot shows this pattern, as well as the overlap in resource use between littoral and pelagic perch (Figure 2). We found a significantly higher SMR in male perch when controlling for habitat and habitat+resource use, but not when controlling for resource use alone (Table 1). We did not see an effect of sex for MMR, AAS, or FAS (Table 1, Table S3). PC2 scores explained 17% of variation and explain mouth direction, length of the second dorsal fin, and tail length (Figure S2). PC3 scores explained 11% of variation and explain differences in mouth direction, body depth, and tail length (Figure S2), which are morphological features that typically differ between littoral and pelagic perch (Svanbäck & Eklöv, 2002). There was no effect of morphology (represented by PC2 and PC3 scores) and no interaction between morphology and habitat in any of our models for any measure of metabolic rate (Tables S1 and S2). We therefore removed morphology from our final models. We found a significant effect of habitat and habitat‐specific resource use on SMR (Table 1; Figure 3). Pelagic perch had a higher SMR than littoral perch, and the pelagic fish with high pelagic resource use had a significantly higher SMR than either of the littoral habitat+resource use groups, which did not differ (Table 2; Figure 3). There was no difference between the SMR of any of our resource use‐only groups, indicating that it is not the prey but a habitat specific aspect driving the difference in metabolic rates between the ecotypes (Tables 1 and 2; Figure 3). We found no significant effect of any explanatory variable on MMR (Table 1; Figure 4). Littoral fish had a significantly higher FAS and AAS, and the habitat‐specific resource use also showed a higher FAS for both littoral‐low and littoral‐high groups compared to the pelagic group (Table 2; Figures 5 and 6).

**TABLE 1:** Output ANOVAs testing the effect of (A) habitat, (B) habitat + pelagic resource use, and (C) pelagic resource use on measures of mass‐independent standard metabolic rate (SMR), maximum metabolic rate (MMR), factorial aerobic scope (FAS), and absolute aerobic scope. Asterisks indicate significance levels (*p < .05, **p < .01, ***p < .001) and bold text indicate the *F*‐value and adjusted *R* ^2^ for each model.

	Factor	SMR	MMR	FAS	AAS
A	Sex	***F***_**(1,47)**_ = 6.46*	*F*_(1,47)_ = 0.03	*F*_(1,47)_ = 2.93	*F*_(1,47)_ = 0.38
	Habitat	***F***_**(1,47)**_ = 31.55***	*F*_(1,47)_ = 1.24	***F***_**(1,47)**_ = 20.48***	***F***_**(1,47)**_ = 4.39*
	Model	*F*_(2,47)_ = 17.11	*F*_(2,47)_ = 0.62	*F*_(2,47)_ = 10.72	*F*_(2,47)_ = 2.24
		*R* ^2^ = 0.40	*R* ^2^ = −0.02	*R* ^2^ = 0.28	*R* ^2^ = 0.05
B	Sex	***F***_**(1,44)**_ = 4.57*	*F*_(1,44)_ = 0.10	*F*_(1,44)_ = 2.51	*F*_(1,44)_ = 0.49
	Habitat + resource	***F***_**(2,44)**_ = 15.70***	*F*_(2,44)_ = 0.66	***F***_**(2,44)**_ = 9.15***	*F*_(2,44)_ = 1.98
	Model	*F*_(3,44)_ = 11.22	*F*_(3,44)_ = 0.44	*F*_(3,44)_ = 6.36	*F*_(3,44)_ = 1.34
		*R* ^2^ = 0.39	*R* ^2^ = −0.04	*R* ^2^ = 0.26	*R* ^2^ = 0.02
C	Sex	*F*_(1,45)_ = 1.31	*F*_(1,45)_ = 0.08	*F*_(1,45)_ = 1.03	*F*_(1,45)_ = 0.30
	Resource use	*F*_(2,45)_ = 0.32	*F*_(2,45)_ = 1.27	*F*_(2,45)_ = 1.81	*F*_(2,45)_ = 1.59
	Model	*F*_(3,45)_ = 0.61	*F*_(3,45)_ = 0.85	*F*_(3,45)_ = 1.37	*F*_(3,45)_ = 1.08
		*R* ^2^ = −0.02	*R* ^2^ = −0.01	*R* ^2^ = 0.02	*R* ^2^ = 0.00

**TABLE 2:** Pairwise comparisons of the estimated marginal means for (A) habitat, (B) habitat + pelagic resource use, and (C) pelagic resource use for measures of mass‐independent standard metabolic rate (SMR), maximum metabolic rate (MMR), factorial aerobic scope (FAS) and absolute aerobic scope (AAS). In the habitat + resource use model, littoral and pelagic refer to the location where the fish were caught, while high and low refer to the pelagic resource use. All significant comparisons are depicted in bold font.

Model	SMR		MMR		FAS		AAS	
	Estimate ± SE	*p*‐value	Estimate ± SE	*p*‐value	Estimate ± SE	*p*‐value	Estimate ± SE	*p*‐value
A. Habitat								
L–P	**−0.062 ± 0.011**	**<.0001**	0.020 ± 0.018	.271	**0.082 ± 0.018**	**<.0001**	**0.051 ± 0.025**	**.042**
B. Habitat + resource use								
LH–LL	0.023 ± 0.017	.372	0.015 ± 0.029	.857	−0.008 ± 0.028	.961	0.013 ± 0.039	.937
LL–PH	**−0.050 ± 0.014**	**.002**	0.027 ± 0.024	.495	**0.078 ± 0.024**	**.005**	0.057 ± 0.032	.185
LH–PH	**−0.073 ± 0.014**	**<.0001**	0.012 ± 0.024	.877	**0.085 ± 0.024**	**.003**	0.044 ± 0.033	.378
C. Pelagic resource use								
Low–Mid	−0.007 ± 0.021	.946	0.028 ± 0.027	.551	0.035 ± 0.031	.505	0.042 ± 0.037	.498
Low–High	−0.014 ± 0.019	.733	0.040 ± 0.025	.259	0.054 ± 0.029	.153	0.061 ± 0.034	.187
Mid–High	−0.008 ± 0.016	.881	0.011 ± 0.021	.857	0.019 ± 0.024	.714	0.018 ± 0.029	.799

## 4 DISCUSSION

Polymorphism in fish is associated with the divergence of several morphological and behavioral traits (Skúlason & Smith, 1995). Our results show that metabolic rates should be considered a part of this larger suite of diverging traits and that future studies should examine the drivers and consequences of metabolic differences between morphs. In our study system, SMR was significantly higher in pelagic compared to littoral perch. However, this variation in metabolic rates does not appear to be correlated with differences in resource use or morphology. Furthermore, because MMR was not related to habitat, the difference in SMR also resulted in a significantly higher aerobic scope in littoral compared to pelagic perch.

We expected that when controlling for morphological factors, such as body depth, we would see a decrease in SMR to compensate for increased drag in deeper‐bodied fish (Pettersson & Brönmark, 1999). Though we did see lower SMR in our littoral fish, which have deeper bodies on average, SMR did not correlate with PC2 or PC3, which are continuous measures of morphology. It is possible that, perch metabolism and morphology evolve in parallel in response to differences in habitat use, since our analyses showed no relationship between these two traits. Our results agree with the finding of studies on Atlantic cod (*Gadus morhua*), yellow perch *(Perca flavescens*), and starry flounder (*Platichthys stellatus*), but not brook trout *(Salvelinus fontinalis)* (Bergstrom et al., 2019; Boily & Magnan, 2002; Reidy et al., 2000; Rouleau et al., 2010). This pattern may indicate that differences in ecological lifestyle, such as locomotor performance, associated with the comparatively high metabolic rates in salmonids also affect the relationship between morphology and metabolism (Killen et al., 2016).

Our analysis of the combined habitat+resource use condition showed differences in SMR between the pelagic fish and the littoral fish from both high and low pelagic resource reliance groups, while there was no significant difference in SMR between the littoral groups (Figure 3b). This pattern suggests that the differences in SMR are not due to differences in foraging strategy since we would expect the littoral‐high fish, which feed more on pelagic resources in the open water, to have an intermediate SMR if that was the case (Figure 3b). Since angling selects for comparatively bold and active individuals (Wilson et al., 2015), future studies could incorporate additional fishing techniques to catch littoral perch with a broader range of behavioral types. This could result in larger differentiation in diet and activity between littoral‐high and littoral‐low groups, allowing for a more thorough examination of the relationship between activity and SMR in our system.

High‐quality fatty acids are found mostly in pelagic prey (Scharnweber, Strandberg, Karlsson, & Eklöv, 2016; Scharnweber, Strandberg, Marklund, & Eklöv, 2016). According to the food‐habits hypothesis (Cruz‐Neto & Bozinovic, 2004; Kim, 2014) we would expect a step‐wise decrease in metabolic rate with the highest metabolic rate in planktivorous fish (high pelagic resource use) and the lowest in benthivorous fish (low pelagic resource use). We however did not find this pattern in resource use groups (Figure 3c). We did see a difference in SMR between male and female fish, and like other studies, male fish had a higher average SMR (Madenjian, 2011). Males and females are subject to different selection pressures, which may be the driver of the observed difference in SMR. This difference in metabolism is in turn linked with variation in life‐history traits between males and females, such as earlier maturation in male fish (Feiner et al., 2017; Höök et al., 2021).

Some possible explanations for why habitat use explained the differences in metabolic rates within our system are: (1) the physical characteristics of the habitats result in plastic changes in metabolic rates due to differences in activity, (2) the natural variation in SMR results in a subset of individuals with high SMR and these individuals selectively utilize pelagic habitats, (3) assortative mating results in the inheritance of genes related to higher metabolic rates in pelagic individuals, or (4) some combination of these mechanisms.

One influential physical characteristic of a habitat is temperature. Though subject to debate, metabolic cold adaptation (Krogh's rule) predicts that cold environments will select for comparatively higher metabolic rates in ectotherms when populations are tested at the same temperature (Pilakouta et al., 2020; Song et al., 2019) but see Steffensen et al. (1994). Pelagic fish in Erken likely encounter cooler temperatures over the summer months because of thermal stratification, even if they do not use cold waters preferentially (Dolson et al., 2009; Kahilainen et al., 2014; Moras et al., 2019; Tunney et al., 2014). Assuming this difference in thermal environments, Krogh's rule offers a potential mechanism for the observed higher SMR in pelagic perch.

Another, and arguably the most prominent, difference between littoral and pelagic habitats is structure. Littoral areas are vegetated and structurally complex, while pelagic habitats are, by definition, open waters. Both complexity itself and the foraging style associated with it are shown to plastically induce changes in perch morphology (Olsson & Eklöv, 2005; Svanbäck & Eklöv, 2006) and may also be important in determining SMR. Interspecific comparisons show that high metabolic rates benefit mobile, visual predators inhabiting structurally simple, fast‐moving, or well‐lit waters, while selection pressure on metabolic rate is relaxed in more benthic species or complex habitats (Auer, Bassar, et al., 2020; Auer, Solowey, et al., 2020; Rosenfeld et al., 2015; Seibel & Drazen, 2007; Webb, 1984). Our results reflect this pattern, though it is unclear whether higher SMR is a plastic response to the open water environment or whether individuals with a high SMR are better able to utilize the pelagic habitat (Auer, Bassar, et al., 2020; Auer, Solowey, et al., 2020), though higher growth rates have been observed following induced differences in feeding mode, supporting plasticity as a mechanism (Olsson & Eklöv, 2005).

The benefit of a higher SMR is increased growth, but this is only true in systems with high food availability (Auer et al., 2016; Burton et al., 2011; Metcalfe et al., 2016). In Erken, age 1+ and 2+ pelagic perch have higher growth rates than their littoral counterparts (Chaguaceda et al., 2020), indicating sufficient food availability for high SMR to be a successful life history strategy in our system. A high AS can also positively affect growth (Auer et al., 2015b; Metcalfe et al., 2016) and is correlated with activity and endurance (Glazier, 2015; Reidy et al., 2000), important aspects of the more active foraging technique used by pelagic perch (Svanbäck & Eklöv, 2003). However, in our study, MMR did not scale with SMR resulting in a lower AS in pelagic perch. This lack of scaling in MMR has also been found under alternate thermal regimes (Sandblom et al., 2016) and food availability (Auer et al., 2016). A small AS may require pelagic fish to make tradeoffs between activities with high aerobic demands, such as foraging and reproduction (Guderley & Pörtner, 2010). With the added stress of increasing water temperatures with climate change, the smaller AS of pelagic perch may help determine the limits for persistence of pelagic perch populations.

Adaptive divergence and the development of distinct morphs can be the first step toward speciation when followed by reproductive isolation, due either to selective mating or physical isolation. Faulks et al. (2015) and Bergek and Björklund (2007) found evidence for assortative mating within littoral and pelagic perch ecotypes and higher kinship values in the pelagic groups (but see Marklund et al., 2019). Though the mechanism facilitating this assortative mating is unknown, differences in timing or spawning depth between ecotypes are seen in coregonids (Hudson et al., 2007) and, along with spawning ground connectedness (Bergek & Björklund, 2007), have been offered as potential mechanisms for assortative mating in perch as well (Faulks et al., 2015). Within coregonids, ecotypes with high activity show over‐expression of genes associated with metabolism (Bernatchez et al., 2010; Jeukens et al., 2010), and future studies on perch could use similar methods to examine whether the differences in SMR between littoral and pelagic fish have a genetic basis.

As with morphology, induced differences in metabolic rate may not be due to a single mechanism and may be caused by an interaction between genes and the environment (Höök et al., 2021; Metcalfe et al., 2016). Pelagic perch from the same Erken population still showed a higher average SMR than littoral perch when maintained under identical lab conditions for a year, though the difference was no longer significant (Figure S3). This shows that perch SMR has a degree of plasticity but suggests that either genetics or conditions during early development play a role in determining SMR since the pelagic group's SMR was still higher at the end of the experiment. Future studies examining what is driving the differences in metabolism between habitats can also help to explain how these differences will interact with climate change. If the differences are driven by metabolic cold adaptation, we may see less differentiation in the future as the duration of the thermocline increases, and hypolimnetic oxygen decreases, limiting access to cooler, deeper waters (Moras et al., 2019). Alternatively, if differences are genetically fixed, and the species is less able to respond plastically to environmental change, the higher SMR of pelagic fish would make them more vulnerable to climate change if their consumption rate or the density of pelagic resources does not scale with their increased SMR. If the differences in SMR are a plastic response to the physical environment, it is unclear how the benefits of a high metabolic rate in structurally simple habitats which require high activity will interact with thermal limitations and temperature acclimation as temperatures increase (Clark et al., 2013; Sandblom et al., 2014). The observed differences across habitats in our study establish this as a valuable system for exploring drivers of intraspecific differences in metabolic rates and examining how the fitness consequences of alternate metabolic phenotypes maintain these patterns in a spatially constrained system.

## AUTHOR CONTRIBUTIONS

**Matilda L Andersson:** Conceptualization (equal); data curation (lead); formal analysis (lead); funding acquisition (equal); investigation (equal); methodology (equal); visualization (lead); writing – original draft (lead); writing – review and editing (equal). **Kristin Scharnweber:** Conceptualization (equal); formal analysis (supporting); investigation (equal); methodology (equal); supervision (equal); visualization (supporting); writing – original draft (supporting); writing – review and editing (equal). **Peter Eklöv:** Conceptualization (equal); formal analysis (supporting); funding acquisition (equal); investigation (supporting); methodology (supporting); supervision (equal); visualization (supporting); writing – original draft (supporting); writing – review and editing (equal).

## CONFLICT OF INTEREST

The authors have no conflicts of interest.

## Supporting information


**Appendix S1** Supporting information

## Data Availability

Data including the AutoResp files for each fish, R scripts used for metabolic rate analysis, and raw data including fish weight, length, time spent in respirometer, and stable isotope values are available on the openly accessible repository Zenodo https://doi.org/10.5281/zenodo.6095875.
