# Supplementary material for: The interaction between metabolic rate, habitat choice, and resource use in a polymorphic freshwater species
Source: Ecol Evol. 2022 Jul 31;12(8):e9129. doi: 10.1002/ece3.9129 (PMC9339753; doi:10.1002/ece3.9129)
Supplement: Supplementary file 1 — Appendix S1 Supporting information [file ECE3-12-e9129-s001.docx]

Supporting information


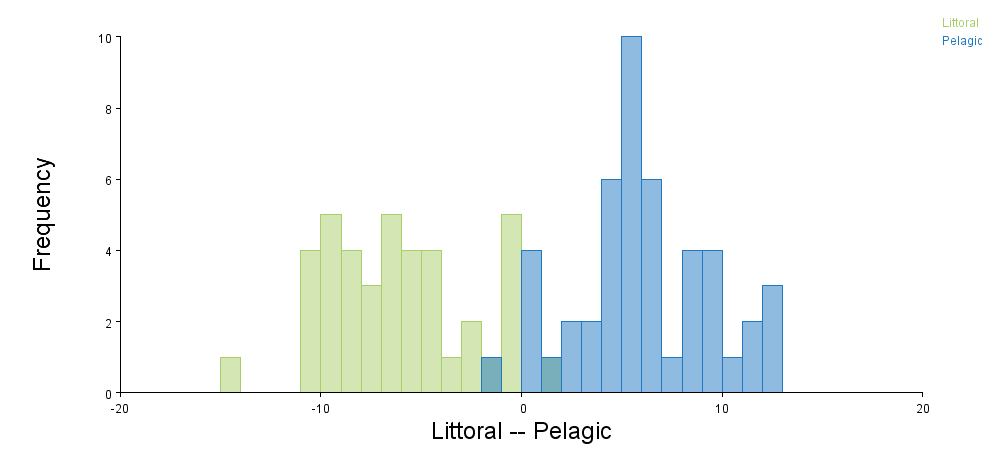


**Figure S1.** Frequency distribution of DFA scores for littoral (green) and pelagic (blue) perch.


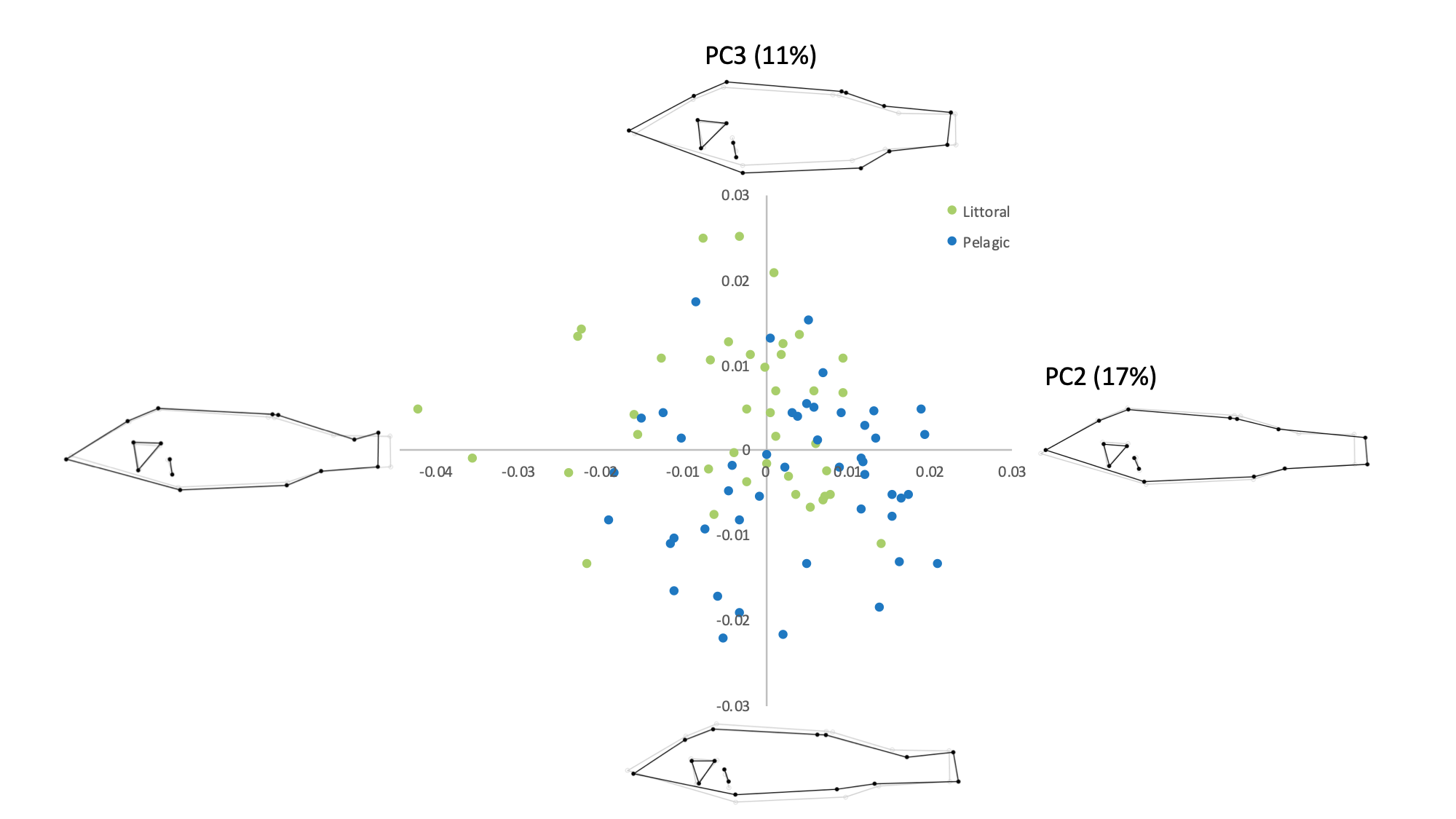


**Figure S2.** Principal component analysis of body shape (PC2 and PC3) in littoral (green) and pelagic (blue) caught perch. The wireframes show body shape changes along the PC axis from -0.075 to 0.075 units of Procrustes distance. Black outlines show the target shape at each extreme, and the grey outlines show the average (mean) shape.

**Figure S3**. The significant difference between weight-independent standard metabolic rate (SMR) of perch maintained in the lab for one month, compared to 11 months. Asterisks indicate significant differences (*p < 0.05, **p < 0.01, ***p < 0.001) based on pairwise comparisons controlling for sex. Perch (n = 28) from the same population and size distribution as those used in our study were maintained in the lab for 11 months for the purpose of another study (Scharnweber et al. in press). After 11 months in the lab, weight-independent SMR of the pelagic perch is still higher than the littoral perch, though the relationship is not significant.

**Table S1**. The output of linear models including morphology (here represented by PC2) testing the effect of A) habitat, B) habitat + pelagic resource use, and C) resource use on measures of mass-independent standard metabolic rate (SMR), maximum metabolic rate (MMR), absolute aerobic scope (AAS) and factorial aerobic scope (FAS). The grey highlight shows the F-value and adjusted R^2^ for each model, and all significant variables are bold.

**Table S2.** The output of linear models including morphology (here represented by PC3) testing the effect of A) habitat, B) habitat + pelagic resource use, and C) resource use on measures of mass-independent standard metabolic rate (SMR), maximum metabolic rate (MMR), absolute aerobic scope (AAS) and factorial aerobic scope (FAS). The grey highlight shows the F-value and adjusted R^2^ for each model, and all significant variables are bold.
